# Investigating the Relationships Between Public Health Literacy and Public Trust in Physicians in China's Control of COVID-19: A Cross-Sectional Study

**DOI:** 10.3389/fpubh.2021.758529

**Published:** 2021-10-28

**Authors:** Dongjin Chen, Qian Zhou, Cornelius B. Pratt, Zhenhua Su, Zheng Gu

**Affiliations:** ^1^Center for Social Governance and Communication, Communication University of Zhejiang, Hangzhou, China; ^2^School of Public Affairs, Zhejiang University, Hangzhou, China; ^3^Lew Klein College of Media and Communication, Temple University, Philadelphia, PA, United States; ^4^College of Media and International Culture, Zhejiang University, Hangzhou, China

**Keywords:** China, COVID-19, health literacy, trust in physicians, agency theory

## Abstract

**Objective:** Public trust in physicians and public health literacy (HL) are important factors that ensure the effectiveness of health-care delivery, particularly that provided during the SARS-CoV-2 pandemic. This study investigates HL as a predictor of public trust in physicians in China's ongoing efforts to control COVID-19.

**Methods:** Data were gathered in February 2020 during the peak of the disease in China. Based on Nutbeam's conceptualization of HL, we measure HL vis-à-vis COVID-19 by using a six-item scale that includes two items each for functional, interactive, and critical HL. Trust in physicians was measured by assessing physicians' capability to diagnose COVID-19. A rank-sum test and ordinal logit regression modeling were used to analyze the data.

**Results:** Two key findings: (a) trust in physician handling of treatment for COVID-19 is reported by about 74% of respondents; and (b) five of the six HL measures are positive predictors of public trust in physician treatment of the disease, with functional HL1 having the highest level of such association (coefficient 0.285, odds ratio 1.33%, *p* < 0.01).

**Conclusions:** Improving public HL is important for better public-physician relationships, as well as for nations' efforts to contain the pandemic, serving as a possible behavioral, non-clinical antidote to COVID-19. Being confronted with the unprecedented virus, humans need trust. Health education and risk communication can improve public compliance with physicians' requirements and build a solid foundation for collective responses.

## Introduction

The urgency and the forthrightness with which a clinical response to the onslaught of COVID-19 was implemented was totemic of the resolve of the worldwide community of interests to ensure global public health. Even so, in the industrialized West, particularly, public protests have been launched, based on the rationales of individual liberty and of freedom of choice, to undermine and defy government measures to control the raging pandemic (1–4). Concerns have also been expressed over the safety and efficacy of some vaccines that are being marketed as critical to protecting the public from COVID-19 (5). From an institutional perspective, one word looms large in the current global response to the public-health impact of SARS-CoV-2: trust (6–10). A burgeoning issue in that context is that China and the rest of the world are confronting a profound crisis of trust in patient-physician relationships (11–15). That mistrust is exacerbated by the evolving COVID-19 pandemic, for which there is a global race to develop and distribute therapeutics and vaccines to protect public health. That race is underscored, particularly in China, by a parallel public-health need: more interventions that target the general public, aiming to improve health literacy and to promote related behavior change (16, 17). In essence, the severity of mistrust in patient-physician relationships and the concerning levels of health literacy could foment discord whenever people are demonstrably anxious about the virus and about their inconveniences from their responses to it. Public response to such a public-health crisis can further undermine efforts by public-health practitioners to control the viral infection and the spread of the disease. It is, therefore, important that public trust in physicians and the health literacy of the public be investigated as essential factors in accessing COVID-19 health-care services and in complying with their health recommendations. The objective of this study, then, is to explore the relationships between HL and public trust in physicians in China vis-à-vis efforts to control SARS-CoV-2 and to treat patients infected with it. In addition to the goal of having better control of the pandemic, the strained public-physician relationships in China merit more attention in part because violence against physicians threatens the country's health-care system. There has been a significant increase in violence against physicians in China (18). Therefore, this study presents suggestions to promote better public-physician relationships.

Trust has been defined as an optimistic relationship between the trustee and the truster (19). Public trust in physicians represents the public's optimistic attitude toward physicians, with the expectation that they will be competent to treat their diseases. Public trust in physicians is a form of professional trust due to their professional competence in medical services (20). The public has a different level of trust in physicians, and we intend to explore the varying degrees of such trust within the context of controlling COVID-19 in China and its relationship with public-health literacy.

Research demonstrates that public trust in medical professionals in China has declined in recent years (21, 22). Various explanations of this troubling trend have been proffered. They include the overarching issue of “inaccessible and unaffordable health care” (*kan bing nan, kan bing gui*) (23); the minuscule patient-physician communication (24); the financial incentives doctors and hospital administrators receive to promote unnecessary health-care services (25); and the experiences of individual patients, such as their satisfaction with previous medical treatment (21). Hsiao argues that patients' limited HL, particularly regarding medical risk, is a major reason for China's medical-related violence (26). Even though patient education has been suggested as a plausible response to combatting this malaise, no study has demonstrated its effectiveness in enhancing public trust in physicians in China (27, 28). This concerning pattern of low trust has been found in other countries as well. In the United States, for example, the introduction of the efficiency-oriented managed-care system has negatively altered patient-physician relationships, and public trust in physicians declined from a high level in the “golden age of doctoring” to a comparatively low-level today (29, 30). This concern over trust is also apparent in Germany (31). In a cross-sectional study, Germans reported significantly less confidence in health-care providers' professional expertise than the British public has in physicians in England, Wales, and the Netherlands (31).

HL is defined as “the cognitive and social skills which determine the motivation and ability of individuals to gain access to, understand and use information in ways that promote and maintain good health” (32) (p. 10). Earlier studies limited the definition to clinical settings, whereas recent research included settings outside hospitals (33), that is, extended to society as a whole. It is critical, then, that research specific to governments' management of COVID-19 be undertaken to ensure, at the microlevel, patient compliance and, at the macrolevel, public safety. It is that lacuna in the extant literature that this work seeks to fill.

Research to date presents conflicting arguments on the potential relationships between them, with findings on both positive and negative associations (34–38). Some researchers have found that higher levels of HL enable patients to be more aware of their own health conditions and to participate in higher-level conversations with physicians, which has led to a deeper sense of trust and better patient-physician relationships (34, 35). In China, cognition- and affect-based trust had a direct positive effect on patient compliance, but internet-health-information seeking had a non-significant impact on patient trust in physicians (39). The authors concluded that seeking internet treatment-related information can improve patient compliance. On the contrary, other studies indicated that high HL, particularly after the emergence of online information, enabled patients to become more knowledgeable and thus more critical of treatments prescribed by their doctors (36, 37). Young, highly educated patients who frequently access online information are regarded as the most critical group and thus are more likely to question doctors' authority rather than be compliant and taciturn (38).

In addition to the two preceding scenarios, there is also the possibility of no association. In other words, it is impossible to find statistical significance between these two variables. For example, self-perceived risk of cardiovascular events was associated with patient follow-up rates; that is, the number of patients who showed up for their clinic appointment suggesting their compliance with physician requests, while patient health literacy did not significantly affect follow-up rates (40). Research findings with regard to the relationship between HL and public trust in physicians are largely dependent on the ethnography of the research. For example, for the positive perceptions literature, hospitals providing inpatient services are a special setting with long-term health-care service (35), and veterans have a greater need to seek multiple medical services (34). Improved HL under these circumstances contributes to a sense of trust because both patients and physicians have more chances to interact with each other, which ultimately reduces misunderstandings between them.

Agency theory and Nutbeam's model of HL guided the development of our framework for investigating the possible association between public trust in physicians and HL. Agency theory enables researchers to understand physician-patient relationships (38, 41). According to the theory, control problems arise from the possibilities of preference discrepancy and information asymmetry between principals and agents (38). In encounters between patients (principals) and physicians (agents), physicians, armed with professional knowledge and experience, prevail in their relationships with patients. Patients, as principals, face their first control problem in their attempts to align physician preferences with those of principals. Skeptics of physician trustworthiness report evidence in physician self-interested behavior, such as the oversupply of services at patients' cost (42). The second control problem arises from information asymmetry, and patients are incapable of monitoring their physicians effectively because of a lack of knowledge. The complexity of medical knowledge and its potential importance for medical treatment make the information gap a very salient issue in the patient-physician relationships. These control disadvantages and patients' perceived vulnerabilities to the threat of diseases nudge them to cede their control over this relationship, making trust a key factor in that relationship (19). However, the proliferation of health-education programs and developments in internet use have resulted in the narrowing of the information gap among some patients (43). The previous physician-dominated relationship evolved into a patient-empowered relationship (38).

Similarly, the definition of HL has also been evolving. Nutbeam identified two approaches to HL: (a) health literacy as a risk factor that needs to be managed, and (b) health literacy as an asset to be built (44). Nutbeam posits that the risk-factor approach in the literature narrows the scope of HL to personal health, mainly occurring in clinical contexts. HL, in this approach, provides individuals with functional knowledge of health care limited to individual literacy and numeracy skills. In the asset approach, individual HL progresses from functional HL to interactive HL and critical HL, providing more empowerment in different healthcare decisions. Interactive HL refers to the capability to apply health information in everyday life and different circumstances; critical HL refers to the capability of analyzing information critically and utilizing that information to exert more control in personal, community, and even social health-care decisions (44) (p. 2075). Nutbeam combines functional HL, interactive HL, and critical HL into a new model of HL for wider application.

Against the preceding backdrop, the purpose of this study is to explore the relationships between HL and public trust in physicians in China vis-à-vis efforts to control SARS-CoV-2. In this study, we hypothesize that public HL will be a positive predictor of public trust in Chinese physicians' control of COVID-19. There are two rationales for this hypothesis. First, the increasing threat of the unprecedented virus has shifted the center of the patient-physician relationship back to the physician side. The literature on patient psychology demonstrates the high level of reliance on physicians for treating severe disease (19, 45). Improving patient knowledge is not likely to challenge physician authority in this circumstance. Second, the control of the pandemic is different from normal health-care services since physicians' opportunistic behavior has been eliminated. The following sections present details on differences between China's control of COVID-19 and normal health care in terms of public trust in physicians. To test the hypothesis, we used a scale of patients' trust in physician knowledge and capability to diagnose and to treat COVID-19 as the dependent variable. For the main independent variable, we used a scale of public HL that includes separate subscales for functional, interactive, and critical HL. A detailed explanation of the measures of the variables is presented in the methods section.

*Main Hypothesis: There will be a significant positive correlation between the level of trust in physicians and an individual's HL level*.

## Background: Public Trust in Physicians During the COVID-19 Pandemic in China

As previously noted, public trust in physicians is not high in China; however, during the management of COVID-19, control measures significantly improved public-physician relationships (46), an outcome that had not occurred since the control of the SARS epidemic in 2003 (47). Public trust in physicians is a dynamic phenomenon, and it varies when certain issues change public attitudes. During COVID-19 in 2020 and the SARS epidemic in 2003, the public witnessed the great sacrifice of medical professionals; that enhanced their trust in physicians.

Post-pandemic, that burgeoning public trust will eventually retreat to pre-pandemic lows. However, these fluctuations in trust have raised serious questions about factors that may be responsible for them. This section includes a delineation of the reasons for an upsweep in public trust of physicians in the COVID-19 pandemic vs. normal circumstances. Using previous models of public trust in physicians or in health-care systems (31, 48), the influences on public trust were divided into three categories: the social conditions, the physician side influences, and the public side influences. The following section was based on those classifications.

### Societal Developments

The health-care system and social media have a significant effect on the public's relationship with the medical community. According to cross-national studies, countries with insurance-based funding for health care have lower public trust in physicians than those with tax-based funding (31). The former type aligns health-care services with commercial transactions and arouses patient consumerist feelings about medical encounters (31). Currently, China has three basic insurance systems: Urban Employee Basic Medical Insurance (UEBMI), Urban Resident Basic Medical Insurance (URBMI), and New Cooperative Medical Scheme (NCMS), which cover more than 98% of the population (49). However, the out-of-pocket payments are exorbitant for many services (50), particularly for low-income patients. Their complaints about unnecessary tests and prescriptions are a reflection of their dim view of the representatives of the health care system—physicians (25). After the COVID-19 outbreak, China's central government decided to foot the bill for all treatment (51), which has done much to allay the public's suspicions regarding physicians' financial motivations.

Meanwhile, the media has contributed to the deterioration of the relationship between physicians and the public (52). China's media comprise both traditional mainstream outlets and new online channels. The idealistic ways in which physicians are portrayed by the mainstream outlets create unrealistically high expectations of them in public. They are depicted as demigods, and these perfect moral images are in direct contrast to the reality that they are just normal people with professional skills (53). In addition, the media also depicted an overly optimistic image of available medical services (52), which causes the public to underestimate the complicated nature and extreme health risks in the real world. Some health-education programs frequently promote the ease of treating severe diseases with simplistic advice from medical experts, especially those in traditional Chinese medicine (54). Unrealistic expectations of physicians are one of many reasons behind this distrust since outrage and indignation are born out of the disillusion of hope (19). Additionally, abundant online information (i.e., Weibo and WeChat, Chinese versions of Facebook and Twitter, respectively) is replete with patients' personal stories of mistreatment that cast doctors in a negative light. Previous studies have documented that net citizens who rely upon online news report a lower level of trust in physicians (55). Therefore, both traditional and online sources are in dire need of stories that portray health-care professionals and medical services in a balanced and realistic way. However, the current dichotomy of good vs. evil in the media only strengthens people's negative perceptions.

Following the outbreak of COVID-19, there has been an increase in the number of positive online comments on health-care professionals (46). Inarguably, their sacrifices in treating COVID patients have earned them praise. Interestingly, positive images have been extended from the traditional media outlets to online sources to the point where negative stories of physicians were hardly ever found on the internet during this period (46). Also, news reports about the disease are unlike those of the pre-pandemic era because the media strives to educate the public about the virulence of the disease and about a new virus that should not be underestimated. People also understand that, even though there are few therapeutic drugs for treating symptoms of the disease, it is plausible that its severity is related to individual immunity (56). Media reports have conveyed a clear public message: that physicians may not have a firm handle on the present health crisis, in contrast to their omnipotent image depicted in pre-pandemic times.

### Developments Among Physicians

The characteristics of physicians are also critical to understanding the public's flaccid trust in them. Research shows that patient-physician relationships are also dependent on doctors' technical knowledge and communication skills (19). Previous studies have suggested that physicians use their discretionary power to create a provider-induced oversupply of health-care services (42), which could lead to patient complaints about unnecessary tests and prescription drugs (57). Chinese physicians have also been reported to have poor communication skills (24). Burdened with heavy workloads, they only allot a few minutes to each patient, exacerbating complaints about their negative professional attitudes.

In the current pandemic, physicians' initiative has been weakened. Because of the overwhelming influx of patients, the pandemic control group in the central government prepared standard procedures to treat patients; that action undermined physicians' standard responsibilities in treating patients with mild symptoms. The Ministry of Health issued seven versions of guidelines on the COVID-19 treatment regimen (58). When treating patients with severe symptoms, physicians were encouraged to make more collective decisions instead of individual ones (59). Further, the treatment of COVID-19 patients was no longer influenced by financial incentives to the degree that the government was making all patient payments. In addition, because of working under several layers of protective gear, it was impossible for them to convey any facial expressions to their patients, and their conversations with patients are also limited because of the concern for self-protection.

### Developments in the Public

Studies indicate that socioeconomic features also influence people's trust in physicians (21, 60, 61). One's age, gender, and socioeconomic class influence one's personal perceptions of doctors, even after controlling for previous experience with them. In addition, people's social trust and their satisfaction with life in general also correlate with their trust in physicians (21, 62). Even though changes in individual traits have been minimal since the outbreak of COVID-19, the public's HL has vastly improved. A cross-sectional study indicates that residents in China have a high level of knowledge about COVID-19 (63), in sharp contrast to their counterparts in countries such as India and the United States (64, 65).

Under normal circumstances, the general public has been criticized for its low level of HL, including having unrealistic expectations of medical treatment and lacking the ability to effectively communicate with physicians during medical encounters (24). During the COVID-19 pandemic, improving personal HL became both an individual and national goal. Previous studies on distrust of physicians were based on the agency theory framework, which highlights the incongruence of preferences and the information asymmetry between patients and physicians (38). The unprecedented nature of COVID has tested the limits of physician knowledge, so people must search for information from alternative sources. Meanwhile, the government waged a nationwide campaign to inspire people to become informed about how to protect themselves from the virus.

Nonetheless, the degree of HL varies widely among the public because of individual differences. In order to assess HL during the COVID-19 pandemic, we combined the public's general HL with their cognitive and critical knowledge of the disease. We adapted Nutbeam's classification to the particular context of COVID-19 and included the following elements of HL with regard to COVID-19: having general health knowledge of the virus, having self-protective measures (functional HL), having the critical skill to process online information on COVID-19 (critical HL), and having the ability to apply specific health information to one's daily life (interactive HL) (57). These dimensions are discussed in the next section.

In sum, the outbreak presents a unique setting for studying public trust in physicians in China. It can be viewed as a natural social experiment in which factors in health-care services have been controlled, allowing us to focus on factors in the public sphere. Without the compounding influences of health insurance systems and of physicians' use of their discretionary power, previously overlooked influences have become prominent, making HL an even more critical factor in the patient-physician relationship.

Previous studies on the relationship between this essential factor and public trust have yielded inconsistent results. HL could be seen as a double-edged sword: infringing on physicians' professional authority while empowering patients and thus pulling the patient-physician relationship in opposite directions. During the COVID-19 pandemic, the positive influences from patients' high HL upon this relationship outweighed negative outcomes from high HL. A comparison of influences underlying public trust before the COVID-19 outbreak demonstrates that public trust is a dynamic phenomenon, varying in accordance with individual characteristics in a unique setting. Therefore, in this study, we focus on individual health literacy.

This social experiment, which mimics a longitudinal study, allowed us to infer that this positive connection is also valid for the cross-sectional study of individuals during the COVID-19 outbreak. In this study, the main hypothesis was that there is a positive correlation between the level of trust in physicians and an individual's HL level, which was then tested with empirical evidence.

## Materials and Methods

### Sampling

A cross-sectional online survey was conducted at the peak of the pandemic from 31 January to 4 February 2020. Because of the pandemic, an online survey is more appropriate and safer than face-to-face surveys. The Institutional Review Board of a major East Coast university in China approved the data-gathering protocol. We used SoJump as a participant-recruitment tool (http://www.sojump.com). SoJump is one of the largest online survey providers in China, with more than 2.6 million registered respondents with different sociodemographic characteristics. The site invited 1,717 randomly selected registered users to participate in an online survey. A total of 1,692 respondents (98.5%) completed the questionnaire. The company used the internal records of registered users to identify potential participants who met three research criteria: (a) have their residence in mainland China, (b) have basic reading and writing skills to complete the survey, and (c) are at least 16 years old. After the final screening, the sample has 1,568 respondents.

Consent to participate was strictly voluntary; no respondent was coerced. Nonetheless, we acknowledge the potential limitations of the sampling and data collection methods used in this study. Specifically, the respondents range from 16 to 74 yr old (M = 32, SD = 10). Education levels ranged from uneducated (0) to those having a Ph.D. or postdoctoral degree (9), with most participants having some degree of a college education. The average monthly household income was between 8001 RMB-10,000 RMB. The survey options included no income (1), 1000 RMB and below (2), 1001 RMB to 3000 RMB (3), 3001 RMB to 5000 RMB (4), 5001 RMB to 8000 RMB (5), 8001 RMB to 10000 RMB (6), 10001 RMB to 15000 RMB (7), 15001 RMB to 20000 RMB (8), 200001 RMB to 50000 RMB (9), and more than 50000 RMB (10). We combine some categories in education and income level, and Table 1 shows the main features of the sample.

**Table 1:** Socioeconomic characteristics of respondents.

	**Proportion (%)**	***M***	***S**D*	
Age (real age)		31.02	9	
16–30	828	24.62	3.77	
31–45	625	35.44	3.89	
46–60	98	51.35	4.11	
61–67	17	62.94	1.75	
Gender				
Male (0)	49.68	–	–	
Female (1)	50.32	–	–	
**Income (RMB)**	**Proportion (%)**			
**Income level** **(scale 1–10)**	**Primary school or below**	**Junior high school**	**High school**	**University or above**
No income	1 (3.03%)	0 (0%)	6 (18.18%)	26 (78.79%)
Less than 1,000	0 (0%)	1 (5%)	3 (15%)	16 (80%)
1,001–3,000	0 (0%)	2 (2.67%)	15 (20%)	58 (77.33%)
3,001–5,000	0 (0%)	2 (1.28%)	24 (15.39%)	130 (83.33%)
5,001–8,000	0 (0%)	7 (2.69%)	24 (9.23%)	229 (88.08%)
8,001–10,000	0 (0%)	3 (1.32%)	12 (5.26%)	213 (93.42%)
10,001–15,000	0 (0%)	2 (0.60%)	8 (2.41%)	322 (96.99%)
15,001–20,000	0 (0%)	0 (0%)	4 (1.62%)	243 (98.38%)
20,001–50,000	0 (0%)	0 (0%)	1 (0.52%)	193 (99.48%)
More than 50,001	0 (0%)	0 (0%)	0 (0%)	23 (100%)

*Data source: Data from the survey were collected during the peak of COVID-19 in China*.

Our survey population could not represent the whole population in China. Our research design is still valid because our main objective is to focus on whether the connection between these two variables is positive or negative. The high proportion of some categories, such as high education levels, will not affect our findings.

### Data Analysis

STATA 14.0 was used to conduct a three-pronged analysis of the data (Stata Corporation, College Station, TX). First, a simple descriptive analysis of the variables was performed. Second, a rank-sum test was used to identify (significant) differences between the levels of control variables and trust in physicians. Third, the measure of trust in physicians was based on a five-point, Likert-type scale of 1 to 5. In other words, these choices are not independent of each other; rather, they are ordinal-level measures. Ordinal logistic regression modeling is used to analyze the relationship between HL and patient trust in physicians.

### Measures

The complexity of the relationships between public trust and public HL lies in the multidimensional nature of both concepts. In extant studies, the predominant classifications include the value dimension and the technical competence dimension, otherwise known as fiduciary and competence trust of physicians (20, 66). Value trust refers to physicians' fiduciary responsibility to patients, while competence trust refers to their technical skills (20, 66). As discussed in section 2, during the COVID-19 pandemic in China, physicians' value trust was unprecedently high because of their dedication to controlling the spread of the virus and the strong pro-doctor propaganda on traditional and online media. Meanwhile, the responses to treating COVID patients have eliminated the potential incentives for physicians to maximize their financial interests because all treatments have been free for patients. Under normal circumstances, public trust in physicians comprises two parts: value trust and competence trust. In the special setting of China's control of COVID-19, this trust has been more about competence trust because of a series of control responses.

Even so, people are still concerned about treatment in light of variations in the technical skills of physicians. During the administration of this survey (February 2020), COVID-19 was still comparatively new to physicians and the public. Confronted with an unprecedented virus, physicians were caught flat-footed. Thus, it was expected that the public would have varying levels of trust toward physicians treating COVID-19. Our measures focused exclusively on physicians' technical competence, particularly as it related to their treating COVID-19.

This study measured respondents' trust in physicians on a single statement (“Because physicians cannot diagnose COVID-19, they are likely to misdiagnose patients because of this lack of knowledge”). The answer is a five-point Likert scale from “strongly disagree” (assigning a value of 5) to “strongly agree” (assigning a value of 1). Previous studies adopted different scales for measuring trust in physicians. Some studies adopted a multi-item scale to measure trust, whereas others also employed a one-item scale to measure trust (21). We used that single- question format because it is the top concern for individuals during the outbreak of COVID-19. Identifying the unprecedented disease from other normal pneumonia is critical to the right treatment, which constructs the most important part of individual evaluation of doctors' medical competence. The measure was reverse-coded so that greater trust was assigned higher values. Previously scholars employed the reverse measure of distrust to calculate the trust level or vice versa (20, 66).

Researchers worldwide have developed HL measures, including the Test of Functional HL in Adults (TOFHLA) in the United States and the Australian Adult Literacy and Life Skills Survey (ALLS) (34). There is no gold standard for assessing HL under normal circumstances (67, 68), and, understandably, there is no agreement on measures to be used during the COVID-19 pandemic. Dumenci and other scholars argued that standard HL measures were more appropriate for primary care services, and for critical diseases, such as cancer, they emphasize that scholars need to develop particular measures (69). We built our measures based on two concerns. The first concern is to build upon previous literature on HL because the structure of health literacy conceptualization should be similar across different diseases. The second concern is to build our measures to reflect the critical elements of health knowledge with regard to COVID-19. We used Nutbeam's model of HL (44, 70), and then divided it into three subscales: health knowledge, self-motivation, and information-processing skills. These categories correspond to functional, interactive, and critical HL under Nutbeam's model of HL. We list two questions for each subscales, and thus, we measure HL using the six-item scale, including two items for functional, interactive, and critical HL, respectively. The questions are listed in Table A1 in the Appendix. Different from comprehensive measures of HL in general, these six questions cover important aspects of HL on COVID-19.

Based on previous research, we also include three important control variables, social trust in general, life satisfaction, and usage of internet news (70, 71). These variables have been used to examine the patient-physician relationship in China. This study explores the validity of the control variables during this pandemic. The measurements for social trust and life satisfaction are listed in Table A1 in the Appendix.

High levels of individual life satisfaction are likely to lead to high levels of trust because optimistic attitudes colors the individual perception of others, including physicians (57). Based on the cross-section analysis, Wang and colleagues find that interpersonal trust is an important predictor for both value trust and competence trust in physicians (72). Also, we measured the frequency of using we-media (i.e., Weibo or WeChat) for news on COVID-19 to measure the extent of reliance on Internet news. We-media refers to the information platform that allows users to receive and send information without the content being screened for accuracy. Demographic characteristics and socioeconomic status were also identified, based on evidence that they are significantly associated with the trust level in physicians in China (21, 71, 72). Table 1 presents the socioeconomic characteristics of respondents.

## Results

### Analysis of Differences in Public Trust in Physicians and in Health Literacy

**Figure 1:**
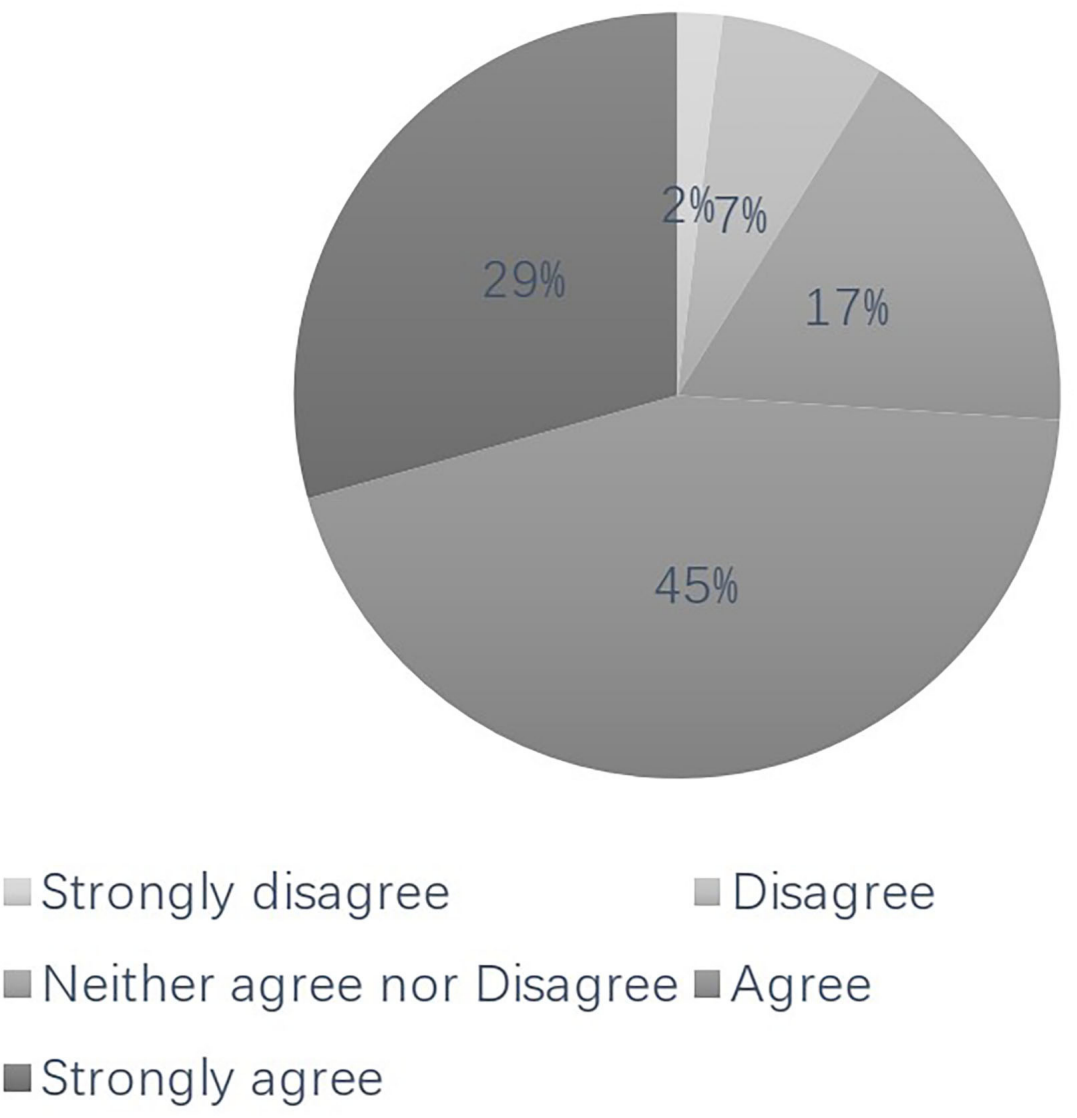
Proportional distribution of respondents based on their trust in physician (%). The distribution of the respondents is clockwise organized from “strongly disagree” to “strongly agree”.

Figure 1 shows the specific distribution of public trust in physicians during the COVID-19 pandemic. Table 2 shows the distribution of HL, social trust, life satisfaction, and we-media usage. Since HL is discussed in relation to COVID-19, the measures yielded in this study differ significantly from standard measures that appear in the existing literature. Furthermore, public trust in physicians examined in this study refers exclusively to the competence of physicians to treat COVID-19 rather than the trust in the existing literature, which includes trust in their personal values or personal responsibilities. It can be seen from Figure 1 that 74% of respondents reported having trust or strong trust in China's physicians, a figure higher than those reported by Zhao and Zhang (21). According to their article, 64.2% of respondents were found to have trust or strong trust in physicians; the mean score of trust in physicians in China was 3.53 in 2016.

**Table 2:** Descriptive statistics for HL and controlling variables.

**Variables**	**Percentage(%)**					***M***	***SD***
	**Strongly Disagree**	**Disagree**	**Neither agree nor disagree**	**Agree**	**Strongly agree**		
Functional HL1^†^	0.89	5.61	9.69	59.57	24.24	4.006	0.803
Functional HL2^#^	0.19	0.26	2.1	16.58	80.87	4.777	0.504
Interactive HL1^#^	1.02	4.15	16.58	48.41	29.85	4.019	0.85
Interactive HL2	0.19	0.7	8.1	59.95	31.06	4.21	0.627
Critical HL1^†^	1.98	9.82	16.01	55.99	16.2	3.746	0.91
Critical HL2^†^	1.53	8.35	14.29	44.84	30.99	3.954	0.961
We-media usage ^#^	9.95	24.68	27.04	25.57	12.76	3.065	1.186
Life satisfaction	5.42	16.26	26.21	38.08	14.03	3.39	1.082
Social trust	1.21	6.7	20.41	60.01	11.67	3.742	0.795

*Data source: Data from the survey were collected during the peak of COVID-19 in China*.

† *Reverse scored. All measurements of these variables are listed in the Appendix, Table A1*.

# *Functional HL2 measurement includes five options, ranging from “no knowledge” (scored as 1) to “full knowledge” (scored as 5). Interactive HL1 measurement includes five options, ranging from “very low capability” (scored as 1) to “very high capability” (scored as 5). We-media usage measurement includes five options, ranging from “never” (scored as 1) to “very often” (scored as 5)*.

With regard to functional health literacy (HL), a reverse-coded measure, 83.8% of respondents (including those who chose “agree” and “strongly agree”) had been educated about how to take preventive measures (functional HL1), and 97.45% of the respondents (including those who chose “much knowledge” and “full knowledge”) had been taught that COVID-19 could be transmitted from person-to-person (functional HL2). As for interactive HL (interactive HL1), only 5.17% of respondents (including those with “low capability” and “very low capability”) were unable to follow their physician's advice from their latest medical appointment, and 0.89% (including those who “disagree” and “strongly disagree”) did not plan to take any preventive measures (interactive HL2).

The critical HL item was reverse coded. 72.19% of respondents (including those who chose “agree” and “strongly agree”) read the information carefully (critical HL1), and 75.83% of respondents (those who selected “agree” and “strongly agree”) carefully analyzed the points of view behind the information (critical HL2). Our findings were consistent with other similar studies on Chinese HL as it relates to COVID-19. For example, Zhong and colleagues found the public's knowledge about COVID-19 to be quite high in China (73), as the correct answer rates were 70.2–98.6%. The results show that public HL during the COVID-19 remains at a high level in China.

### The Association Among Trust in Physicians and the Control Variables

A rank-sum test was used to compare the differences of control variables and the results, listed in Table 3. In order to save space for this table, we combine some categories when we perform rank-sum tests. For example, all respondents are divided into two groups based on their ages. The proportion of the first group (aging 16–41) among all respondents is 88.52%, 11.48% for the second group (aging 42–67). The rank-sum and *P* values are shown in Table 3. There were no significant associations between trust in physicians and sociodemographic characteristics (*p* > 0.05 for age, gender, income, and education). As for the other control variables, we-media usage and high social trust were found to have no significant correlation with trust in physicians (*p* > 0.05). Confidence in medical staff was associated with life satisfaction (*p* < 0.01) and social trust (*p* < 0.1). In other words, those who were satisfied with their current life circumstances and trusted others more maintained a higher level of trust in physicians.

**Table 3:** Association between trust in physicians and control variables.

**Parameters**	**Category**	**Proportion among all respondents (%)**	**Rank Sum**	***p***
Age^†^	16~41	88.52	606,489	0.130
	42~67	11.48	66,892	
Gender	Female	49.68	342,732	0.442
	Male	50.32	330,648	
Monthly household income ^‡^	<10,000 RMB	49.23	326,867	0.182
	≥10,000 RMB	50.77	346,504	
Education level^§^	Primary school	0.06	0	0.239
	Junior high school	1.08	4,665	
	High school	6.19	37,270	
	University or higher	92.67	631,445	
We-media usage	Never	9.95	62,226	0.148
	Seldom	24.68	165,650	
	A couple of times	27.04	178,981	
	Multiple times	25.57	179,052	
	Very often	12.76	87,471	
Life satisfaction	Strongly disagree	5.42	28,316	0.002^***^
	Disagree	16.26	105,793	
	Neither agree nor disagree	26.21	156,153	
	Agree	38.07	267,141	
	Strongly agree	14.03	115,979	
Social trust for others	Strongly disagree	1.21	7,336	0.056^*^
	Disagree	6.7	33,452	
	Neither agree nor disagree	20.41	119,559	
	Agree	60.01	425,334	
	Strongly agree	11.67	87,701	

† *To save space on the table, respondent ages were categorized into two groups for the rank-sum test*.

‡ *Respondents' monthly household income are categorized into two groups for rank-sum analysis*.

§ *To save space on the table, we divided the respondents' education levels into four groups for the rank-sum test*.

*** *p < 0.01;*

** *p < 0.05;*

* *p < 0.1*.

### Ordinal Logistic Analyses Results

Finally, an ordinal logistic model was developed to analyze the data. Before modeling, we conducted collinearity diagnoses. Through the Collin test, the values of VIF and Tolerance can be observed. Each VIF value is less than 5, and the Tolerance values are greater than 0.1, indicating that there is no collinearity among the variables; that is, the results of the key model are reliable. The result of the Collin test is reported in Table A2 in the Appendix. Results of the ordinal logistic model in Table 4 are from Model 1 and provide substantial support for the hypothesis that HL has a positive relationship with trust in physicians. Thus, it is clear that functional HL significantly improves public trust in physicians (functional HL 1 coefficient 0.302, the odds ratio between the reference group and the compare group is 1.353%, *p* < 0.01; functional HL 2 coefficient 0.158, the odds ratio between the reference group and the compare group is 1.171%, *p* < 0.1). In addition, the effect of interactive HL on the public's trust in physicians is significant (interactive HL 1 coefficient 0.209, the odds ratio between the reference group and the compare group is 1.232%, *p* < 0.1; interactive HL 2 coefficient 0.237, the odds ratio between the reference group and the compare group is 1.267 %, *p* < 0.01). Furthermore, critical HL also significantly improves public trust in physicians (critical HL 1 coefficient 0.148, the odds ratio between the reference group and the compare group is 1.160%, *p* < 0.05; critical HL 2 coefficient 0.202, the odds ratio between the reference group and the compare group is 1.224%, *p* < 0.01). In Model 2, in which we-media, life satisfaction, and social trust were added as control variables based on Model 1; the results continue to show that HL can promote public trust in physicians. Also, we-media exerts no influence on one's trust in physicians. However, life satisfaction (coefficient 0.106, the odds ratio between the reference group and the compare group is 1.112%, *p* < 0.05)and social trust (coefficient 0.141, the odds ratio between the reference group and the compare group is 1.151%, *p* < 0.05) have a significant influence on public trust in physicians. When controlling for socio-demographic variables according to Model 3, HL can still significantly affect public trust, with the exception of functional HL 2. Since this variable was found to be significant in both Models 1 and 2, it would not affect the overall influence of HL on trust in physicians. Among the six measures of HL, functional HL 1 was found to be the major predictor, according to Model 3 (coefficient 0.285, the odds ratio between the reference group and the compare group is 1.33%, *p* < 0.01). Hence, the main hypothesis in this study is supported.

**Table 4:** Ordinal logistic regression analysis of health literacy and trust in physicians.

	**Trust in Physicians**		
	**Model 1**	**Model 2**	**Model 3**
Functional HL 1	0.302 (0.063)^***^	0.288 (0.063)^***^	0.285 (0.064)^***^
Functional HL 2	0.158 (0.094)^*^	0.165 (0.094)^*^	0.138 (0.095)
Interactive HL1	0.209 (0.059)^***^	0.168 (0.060)^***^	0.160 (0.060)^***^
Interactive HL2	0.237 (0.083)^***^	0.197 (0.084)^**^	0.239 (0.085)^***^
Critical HL 2	0.148 (0.058)^**^	0.141 (0.059)^**^	0.121 (0.059)^**^
Critical HL 2	0.202 (0.055)^***^	0.196 (0.055)^***^	0.203 (0.055)^***^
We-media Usage		0.014 (0.040)	0.006 (0.040)
Life satisfaction		0.106 (0.046)^**^	0.126 (0.047)^***^
Social trust		0.141 (0.063)^**^	0.158 (0.063)^**^
Age			−0.023 (0.006)^***^
Gender			−0.051 (0.095)
Income			0.022 (0.027)
Education level			−0.023 (0.057)
*N*	1568	1568	1568

*** *p < 0.01;*

** *p < 0.05;*

* *p < 0.1*.

Since we only use a single question to measure the trust in physicians, we add two additional measures to further test the relationship in order to ensure the robustness of the results. Variable One is based on the measure of the answer to the question “Do you agree that the hospital's diagnosis of COVID-19 is highly accurate” and Variable Two is “Do you think the professionalism of the scientists involved in the prevention and treatment of COVID-19 is convincing”. There are five options, from “completely disagree” to “completely agree.” We use these two measures as approximate measures of the public trust in physicians, one representing an aggregation of physicians and the other representing their professionalism. The results are similar to our findings in Table 4, and are reported in Table A3 in the Appendix.

As for the sociodemographic variables tested in Model 3, the variable of age was shown to be negatively related to trust in physicians. Thus, the older one gets, the less one trusts physicians. Gender, income, and education have no significant effect on one's trust in physicians.

Our sample included more educated people than the general population in China, which would affect our results' generalization. The online survey is more popular among highly educated individuals because they are more capable of using mobile phones. Since there exists a possibility that the relationship between public trust in physicians and public HL (health literacy) may vary among the different populations, we divided the respondents into two subgroups, that is, the less educated and the more educated. The more educated subgroup includes respondents with high education and above. We compared the coefficients and significance of six measures of public HL in the modeling of trust in physicians between these two subgroups. We found that the relationship between trust in physicians and HL in the low-educated subgroup was not significant, while the relationship in the high-educated subgroup is almost significant. Five out of six measures of HL have significant positive relationships with trust in physicians; the coefficients of four out of six HL measures are larger than the counterparts in the lower educated subgroup. These results indicate that the selection bias would overestimate the regression estimates between pubic trust in physicians and public HL. The conclusions of the data in this article are more suitable for populations with higher education. We reported our results of these two modeling in Table A4 in the Appendix.

## Discussion

Patient-physician relationship has never been more important in the background of the global pandemic. The information gap raised in the agency theory framework suggests that a growing need among patients for health information coexists with more self-awareness. In China's control of COVID−19, this trend upends the traditional balance of the patient-physician relationship. There is a discussion on the relationship between patients' HL and their trust in physicians in normal times (57, 72). This study investigates public HL as a predictor of public trust in physicians in China's pandemic control of COVID-19. Based on agency theory and on Nutbeam's model of HL, we conclude that the uniqueness of the setting mediates the relationship between them. In the context of China's control of the pandemic, control regulations modified patient-physician relations and the impact of public HL on the trust in physicians.

Our results demonstrate significant positive relationships between HL and public trust in physicians, providing empirical evidence for the main hypothesis. In this study, trust in physicians was treated as a one-dimensional concept that was limited to physician competence. HL was viewed as a multifaceted concept that includes health knowledge, self-motivation, and information processing skills. During the COVID-19 pandemic, the positive connection between these two elements was revealed in a series of statistically significant correlations, shown in Table 4. Based on these results, a causal relationship with the vector from HL to public trust could not be determined. Among these three dimensions, functional knowledge to recognize the highly infectious nature of the virus might be based on following medical experts' suggestions in the media. With regard to communicative literacy, respondents with high awareness of their health problems and high willingness to adhere to previous advice from their doctors demonstrate prior compliance. Placing their trust in physicians' treating COVID-19 can be perceived as an extension of their ongoing confidence in their personal physicians. Better functional knowledge and communicative literacy have been already proven to have a strong correlation with a high-quality patient-physician relationship (24, 74).

The only exception to this dyadic interaction might be critical literacy because trust in physicians may not have a causal effect on one's critical literacy and online information processing skills; however, this component has been shown to have a possible negative relationship with public trust in previous studies (75, 76). The discrepancy between patients' perceptions of health issues based on online sources and doctors' perceptions weakens the traditional paternalistic position of physicians (38). However, some scholars argue that seeking quality online health information could improve patient trust in physicians because this information helps build interdependence between patients and physicians (76). During the COVID-19 pandemic, people also confronted “infodemic,” a term used to describe the fake news common on online platforms (77). That makes having a high level of critical HL particularly important. People with low levels are more likely to be swayed by rumors and paranoia, placing them further away from science-oriented information. However, those with high critical literacy are more likely to trust the representatives of medical science, physicians. This study provides more evidence for the current academic debate on the relationship between HL and public trust in physicians. The positive role of HL in promoting better public-physician relationships has been confirmed during the COVID-19 pandemic based on the empirical analysis discussed above.

The division of value trust and competency trust within this unique setting has enabled us to reach a better understanding of their connections. China's control of the COVID-19 pandemic can be perceived as a large-scale social experiment in which the element of value trust is controlled by a series of governmental interventions, as discussed in Section 2. A possible explanation for the inconsistencies in previous studies of HL and public trust may be attributed to the multi-dimensional nature of these concepts. Our conclusion can be used to study the connection between individual HL and public trust in other settings, and the relevant research design can be utilized to examine the multi-dimensional nature of these concepts.

With regard to control variables, our results are mixed in comparison to those of existing studies. For example, our finding that social trust and satisfaction have positive impacts on individual trust in physicians is consistent with those of previous studies (57, 72). This generic trust strengthens personal contact with other people and social organizations, of which physicians are certainly an important part. In addition, individuals' satisfaction with their current lives might color their perception of others and cause them to have a more positive attitude toward them.

However, some of the results run counter to generally agreed-upon beliefs in previous studies. Existing studies on public trust reached a consensus that elderly people are more likely to have a high level of trust in physicians because of their multiple encounters with them (19). This long-term relationship is generally thought to build a positive reciprocal relationship. However, in COVID-19 settings, we found that age has a negative relationship with trust. Based on the current statistics on the demographic character of infected patients, older people are more likely to develop serious symptoms (78); therefore, they are more cautious about the possibility of malpractice. Thus, they are more vigilant about choosing physicians to treat COVID-19.

Another finding that is not aligned with previous research is the influence of people's use of the internet to receive news and information on the pandemic. Research has found that people who rely on the internet for news are more likely to have lower trust in physicians because there are inaccurate accounts on treatment (71). Our findings indicate that internet use does not significantly affect trust levels. During the COVID-19 pandemic, negative online news about physicians lessened to the degree that its impact upon public trust disappeared.

The COVID focus of this study does not diminish the validity of its findings, which may enhance our understanding of patient-physician relationships in other clinical settings in China and in other countries. As we note in Section 2, this pandemic unexpectedly provides a social experiment to observe China's health-care services. China's ongoing experience is a large-scale social experiment in which financial, media, physician discretion and other factors have been controlled. Better patient-physician relationships are based on more patient education. Studies find empirical support for agency theory in normal times (38), and our study demonstrates its validity in a global pandemic, an unusual period.

Our main research question is on the analysis of the relationship between public trust in physicians and public-health literacy in China's control of COVID-19. Even though this relationship is for a particular occasion, the findings from this special occasion provide a rare opportunity to explore this relationship in a straight and direct way. Current studies on the relationship between public trust in physicians and public health literacy could hardly control macro-level influences such as the payment system of medical services. These influences could complicate the relationship, and scholars turn to certain research designs (cross-nation studies) to identify the possible influence (20). China's control of COVID-19 creates a de-facto social experiment, where many macro-level influences have been controlled. Thus, we could find a comparatively straightforward way to focus on individuals' properties only and explore the relationship between their trust in physicians and their health literacy.

However, the particular setting leads to a concern about the generalization of our findings, especially about the validity and reliability of the measurements of basic concepts. We try to make a balance between two considerations. The first is to keep our studies consistent with previous studies, including the selection of variables and their measurements. For example, we employed Nutbeams' structure of conceptualization of HL to design our measures of HL. The second is to take into account the specific situation in China's control of COVID-19, which is unprecedented. We identified critical elements of the public attitudes toward treating and protecting from COVID-19 and integrated them into our measurements. By combining these two considerations, we believe our findings could be expanded to understand the relationship between public trust in physicians and HL in normal times.

This finding can also be supported by the public's self-protection behavior in China, among which wearing facial masks is the most prominent one. In China, the highly-disciplined behavior is born out of two important conditions: their knowledge of the virus derived from all forms of media and their trust in physician counseling regarding the use of facial masks. In China, there is an agreement that wearing mask is the easiest way to protect oneself, eliminating all kinds of distorting “noises” that might undermine public compliance. Its authoritarian style of fighting the virus has resulted in a lot of controversies in the world (79). Among China's control strategies, hard-control measures, such as lockdown measures, are more inconvenient than other soft-control ones, such as wearing facial masks. Wearing facial masks is an important control strategy in China, but it does not mean that it is an infringement on individual freedoms, as viewed in some industrialized nations. Encumbered with an unusual enemy in the virus, the public should have more trust in their health-care professionals and in fellow citizens.

These findings have several implications for controlling the current pandemic worldwide, as well as for improving patient-physician relationships in China. The study suggests that having a high level of health literacy bolsters one's trust in physicians, particularly trust in physician competence, which is in itself likely to promote more compliance, as the control of the pandemic in China demonstrates. Martin emphasizes that high levels of trust in physicians is part of “societies' reserves of generalized trust” (20). Encumbered with an unprecedented health-care crisis, social trust has never been so important.

The tense public-physician relationship is among a series of strained social relationships, enabled by partisan politics (i.e., the United States) and international conflicts (i.e., W.H.O.). Yet, the importance of trust in physicians reminds us that solidarity in collective actions and individual responsibility are vital to fighting the virus. Groups need to set aside their ideological and political differences in the ongoing battle against a global scourge.

Based on our findings, we also have suggestions for improving the patient-physician relationship in China. The public needs to expand its HL and avoid solely relying on physicians–a dereliction of personal duties and responsibilities. This is not an easy task because it requires the accumulation of health information, the ability to apply it in one's daily life, and the acquisition of critical skills in distinguishing between accurate and inaccurate information. Physicians can direct patients to reliable sources of health information. China's media could also positively contribute by providing more programs on medical risks, thus offering people more reasonable expectations about health care.

Our study has several limitations. First, the sampling may not be adequately representative, given the high level of education among respondents. That may be an artifact of selection bias. That means the estimates of our study are more appropriate for the population with similar characteristics. We can improve our further understandings by two potential solutions. The first is to conduct more rigorously designed surveys targeting the general public, and the other is to focus on respondents with low education levels. Second, measures on respondents' HL level are designed for COVID-19 only and could hardly be expanded to other diseases. Third, public trust in physicians for treating COVID-19 may be different from a general trust as well as patients' trust in their personal physicians. Finally, future studies should include more in-depth, one-on-one discussions on mechanisms of improving HL.

## Conclusion

This study demonstrates that public HL and trust during the breakout of COVID-19 are high. And we also find that public HL is a positive predictor of trust in physicians during this unusual period. This finding adds significant evidence to current discussions on the potential relationship between HI and public trust in physicians. As long as physicians' opportunistic behavior is mitigated, the level of patients' trust in physicians is positively associated with their HL. Besides, our finding has practical significance even though the uniqueness of China's COVID-control measures may not be replicated elsewhere. First, the global COVID pandemic is not likely to disappear in the near future. Following physician suggestions and taking action to ensure self-protection—e.g., getting vaccinated and wearing facial coverings—are key responses to slowing or stopping the spread of the virus. Second, in the context of a strained patient-physician relationship in China, improving patients' HL results in a higher level of trust in physician competence, which lends empirical support to using more health-education and risk-communication interventions. For both normal occasions and pandemic settings, health education and risk communication are indispensable to collective actions against diseases.

## Data Availability Statement

The raw data supporting the conclusions of this article will be made available by the authors, without undue reservation.

## Ethics Statement

The studies involving human participants were reviewed and approved by the Ethics Committee of Zhejiang University. The patients/participants provided their written informed consent to participate in this study.

## Author Contributions

DC and ZG: conceptualization and methodology. DC and QZ: software. DC and ZS: formal analysis. DC, CP, and QZ: writing—original draft preparation. DC, QZ, CP, ZS, and ZG: writing—review and editing. QZ: visualization. DC: supervision, project administration, and funding acquisition. All authors contributed to the article and approved the submitted version.

## Funding

This work was supported by the General Project of Zhejiang Philosophy and Social Science Fund, A study on using health communication empowerment to improve health-care governance in Zhejiang (No: 21NDJC111YB).

## Conflict of Interest

The authors declare that the research was conducted in the absence of any commercial or financial relationships that could be construed as a potential conflict of interest.

## Publisher's Note

All claims expressed in this article are solely those of the authors and do not necessarily represent those of their affiliated organizations, or those of the publisher, the editors and the reviewers. Any product that may be evaluated in this article, or claim that may be made by its manufacturer, is not guaranteed or endorsed by the publisher.

## Abbreviations

HL: Health Literacy
UEBMI: Urban Employee Basic Medical Insurance
URBMI: Urban Resident Basic Medical Insurance
NCMS: New Cooperative Medical Scheme
TOFHLA: Test of Functional Health Literacy in Adults
ALLS: Adult Literacy and Life Skills Survey.
