# Supplementary material for: Investigating the Relationships Between Public Health Literacy and Public Trust in Physicians in China's Control of COVID-19: A Cross-Sectional Study
Source: Front Public Health. 2021 Oct 28;9:758529. doi: 10.3389/fpubh.2021.758529 (PMC8584494; doi:10.3389/fpubh.2021.758529)
Supplement: Supplementary file 1 [file Appendix.docx]

**Appendix**

| Table A1: Measurement Instrument | |
| --- | --- |
| Construct | Items |
| Functional HL1^†^ | I don’t know what protective measures should be taken. |
| Functional HL2^#^ | The new coronavirus has the ability to spread from person-to-person. |
| Interactive HL1^#^ | I understand my diagnosis and am able to follow my doctor’s advice. |
| Interactive HL2 | After learning about COVID-19, I plan to take action to prevent its spread. |
| Critical HL1^†^ | I only browse information about COVID-19 at random. |
| Critical HL2^†^ | I didn’t carefully consider opinions about the epidemic. |
| We-media usage^#^ | During the past week, how often did you obtain information about COVID-19 through We-media? |
| Life satisfaction | I am satisfied with my current life. |
| Social trust | Overall, a majority of people in society are trustworthy. |

Data source: the data from the survey was collected during the peak of COVID-19 in China.

Note: ^†^ Reverse scored, so that low values represent low literacy, low trust or low satisfaction.

^#^ Functional HL2 measurement includes five options, ranging from “no knowledge” (scored as 1) to “full knowledge” (scored as 5). Interactive HL1 measurement includes five options, ranging from “very low capability” (scored as 1) to “very high capability” (scored as 5).We-media usage measurement includes five options, ranging from “never” (scored as 1) to “very often” (scored as 5).

Table A2. Collinearity Diagnosis

| Variable | VIF | SQRT VIF | Tolerance | *R^2^* |
| --- | --- | --- | --- | --- |
| Trust in physicians | 1.10 | 1.05 | 0.9058 | 0.0942 |
| Functional HL 1 | 1.18 | 1.08 | 0.8507 | 0.1493 |
| Functional HL 2 | 1.05 | 1.03 | 0.9492 | 0.0508 |
| Interactive HL1 | 1.15 | 1.07 | 0.8688 | 0.1312 |
| Interactive HL2 | 1.23 | 1.11 | 0.8154 | 0.1846 |
| Critical HL 2 | 1.25 | 1.12 | 0.8001 | 0.1999 |
| Critical HL 2 | 1.25 | 1.12 | 0.7981 | 0.2019 |
| We-media Usage | 1.02 | 1.01 | 0.9835 | 0.0165 |
| Life Satisfaction | 1.13 | 1.06 | 0.8840 | 0.1160 |
| Social Trust | 1.11 | 1.06 | 0.8970 | 0.1030 |
| Age | 1.22 | 1.10 | 0.8195 | 0.1805 |
| Gender | 1.03 | 1.01 | 0.9712 | 0.0288 |
| Income | 1.30 | 1.14 | 0.7685 | 0.2315 |
| Education Level | 1.20 | 1.10 | 0.8338 | 0.1662 |

Table A3. Robust Test Using Similar Measures of Trust in Physicians.

|  | Trust in Physicians | |
| --- | --- | --- |
|  | Model 1 | Model 2 |
| Functional HL 1 | 0.225(0.070) *** | 0.058(0.068) |
| Functional HL 2 | 0.231(0.104) ** | 0.522(0.099) *** |
| Interactive HL1 | 0.162(0.064) ** | 0.154(0.064) ** |
| Interactive HL2 | 0.146(0.091) | 0.388(0.089) *** |
| Critical HL 2 | 0.250(0.065) *** | 0.001(0.062) |
| Critical HL 2 | 0.203(0.060) *** | 0.171(0.059) *** |
| We-media Usage | -0.127(0.043) | -0.039(0.043) |
| Life Satisfaction | -0.038(0.050) | 0.140(0.050) *** |
| Social Trust | 0.094(0.068) | 0.311(0.067) *** |
| Age | 0.008(0.006) *** | -0.014(0.006) ** |
| Gender | -0.052(0.103) | -0.131(0.101) |
| Income | 0.039(0.029) | -0.055(0.029) * |
| Education Level | 0.039(0.060) | 0.028(0.061) |
| *N* | 1568 | 1568 |

Note:

Model 1 uses Variable One as the dependent variable.

Model 2 uses Variable Two as the dependent variable.

*** *p < 0.01; ** p < .05; * p < 0.1.*

| Table A4: Modeling in two different educated subgroups | | |
| --- | --- | --- |
|  | Trust in physicians | |
|  | Less educated | More educated |
| Functional HL 1 | -0.002(0.131) | 0.366***(0.073) |
| Functional HL 2 | 0.179(0.178) | 0.181(0.113) |
| Interactive HL1 | 0.189(0.128) | 0.157**(0.068) |
| Interactive HL2 | 0.146(0.174) | 0.242**(0.098) |
| Critical HL 2 | 0.146(0.120) | 0.117*(0.068) |
| Critical HL 2 | 0.159(0.119) | 0.227***(0.063) |
| We-media Usage | 0.082(0.085) | -0.009(0.046) |
| Life Satisfaction | 0.128(0.100) | 0.127**(0.053) |
| Social Trust | 0.068(0.145) | 0.185***(0.071) |
| Age | -0.005(0.009) | -0.034***(0.008) |
| Gender | 0.169(0.212) | -0.097(0.108) |
| Income | 0.067(0.058) | 0.006(0.031) |
| *N* | 331 | 1237 |

Note: *** *p < 0.01; ** p < .05; * p < 0.1.*
